# Editorial: Nervous system and reproduction: a highly integrative partnership

**DOI:** 10.3389/fendo.2025.1621411

**Published:** 2025-05-21

**Authors:** Hernan E. Lara, Mauricio D. Dorfman, Artur Mayerhofer

**Affiliations:** ^1^ University of Chile, Santiago, Chile; ^2^ University of Washington, Seattle, WA, United States; ^3^ Ludwig Maximilian University, Munich, Germany

**Keywords:** hippocampus, hypothalamic–pituitary–gonadal axis, ovary, testis, nervous system

Reproductive success in mammals requires mechanisms that synchronize and regulate the reproductive system and, furthermore, the interaction between males and females. Central to this is the hypothalamus, which regulates the pituitary, which in turn governs gonadal functions, including steroid production. Steroids regulate reproductive organs and feed back to the hypothalamus. However, hormones do not act alone but rather in concert with a well-developed neural network.

Over 40 years ago, Kawakami et al. (1) first documented a neural pathway originating in the hypothalamic paraventricular nucleus (PVN), which reaches the gonads. Gerendai et al. (2), using trans-neuronal retroviral technology, demonstrated a sympathetic pathway between the PVN and the ovary. A similar organization was found in males. Such neuronal pathways likely allow both efferent influences and afferent, sensory feedback signaling.

Since then, it became clear that next to sensory signals from the periphery, also metabolic signals and input from areas of the brain involved in emotion and memory, as well as stress regulation, converge at the level of the hypothalamus and influence the efferent neuronal signaling to the periphery and the gonads.

This Research Topic aimed to examine such aspects.

One of the emerging questions in neuroendocrinology is how emotions and memory are linked to reproductive success. The article by Castillo et al. offers new insights. Almost all types of sensory information trigger activation of the hippocampus, which in turn distributes output signals to the hypothalamus and other parts of the diencephalon. This study suggests a neural projection from the hippocampus to the hypothalamus, which is involved in the regulation of reproductive function. The hippocampus, a crucial center for memory, may thus serve as an integration hub for combined hormonal and cognitive function. The hippocampus, importantly, may override the gonadotropic control of the gonads by acting via neural connections to the gonads. This reveals a novel mechanism by which learning processes are linked to the neuroendocrine regulation of ovarian functions.

Hypothalamic PVN-derived corticotrophin-releasing hormone (CRH) plays a major role in regulating the stress response. Regarding the integration of neural and hormonal responses, Yu et al. explored, how reproduction is influenced by the stress responses and the PVN. However, whether stress influences reproductive functions by modulating PVN-derived CRH or the hypothalamic–pituitary–adrenal (HPA) axis, remains to be determined. It is also possible that stress activates sympathetic nerves originating in the magnocellular region of the PVN. Yu et al. propose that CRH neurons in the PVN play a functional role in disrupting ovarian cyclicity and the preovulatory LH surge.

The observation that the activity of the gonadotrophin-releasing hormone (GnRH) pulse generator during chronic stress maintains its activity during certain phases of the estrous cycle opens the possibility that locally produced pituitary regulators may act as strong modulators of reproduction. The study by Wang et al. supports this possibility in sheep. Their analysis of the Booroola fecundity mutation (FecB) in Small Tail Han sheep, which enhances ovulation rates and litter sizes, points to an effect on the hypothalamic–pituitary–gonadal (HPG) axis. They identified GABA as a potential regulatory factor within the HPG axis modulating GnRH and gonadotropin feedback loops.

In recent years, several local regulators, active at the HPA level, were identified. They include neuroactive peptides, discovered using bioinformatics, such as Spexin (SPX, NPQ), a 14-amino acid neuroactive peptide (Chen et al.). Its amino acid sequence is highly conserved across species. It acts as a neuromodulator inhibiting reproductive performance and regulating other functions associated with reproduction, including feeding behavior, obesity, glucose and lipid metabolism, and stress. Of note, the *spx* and *kiss1* genes are located on the same chromosome and share similarities in their peptide sequences. The close relationship with kisspeptin, a widely studied peptide involved in metabolism and reproductive function, underscores the interconnected roles of neuromodulators acting in the CNS and controlling the peripheral reproductive regulation.

Metabolic signals (including, e.g., leptin) are mainly integrated in the hypothalamus, where they regulate both energy balance and reproductive function. Astudillo-Guerrero et al. propose that leptin may play a role in regulating the activity of the sympathetic innervation of reproductive tissues. While obesity and its metabolic complications are often linked to ovarian dysfunction, the mechanisms underlying this connection remain unclear. This review discusses the pieces of evidence that imply a crucial role of metabolic signals, including nutrients and hormones, such as leptin, insulin, and glucagon like peptide-1 (GLP-1), which act directly on the hypothalamus, in the modulation of sympathetic innervation of the ovary and other reproductive organs. Hyperactivation of sympathetic nerves in adulthood, due to early-life metabolic programming, could thus be a possible cause of reproductive and metabolic disorders such as polycystic ovary syndrome.

The minireview by Frungieri and Mayerhofer summarizes the roles of biogenic amines in the regulation of the male gonad. Their influence is documented at the level of the CNS and, locally, at the level of the testis. Thus, catecholamines have a role in the regulation of GnRH secretion and the HPA. A descending multi-synaptic, pituitary-independent neural pathway connecting the hypothalamus with the testis in the rat involves beta-adrenergic receptors. GnRH neurons also express histamine receptors, and synaptic contacts between serotoninergic neurons and GnRH neurons were described. Pineal-derived melatonin, by binding to the suprachiasmatic nucleus and the pars tuberalis, and via modulation of kisspeptin signaling, also influence GnRH and gonadotropins. Besides such central actions, biogenic amines play a number of local roles in the male gonad, where they, e.g., regulate steroid production. These are described in this minireview in detail, and knowledge gaps are pinpointed.

## Concluding remarks

**Figure 1:**
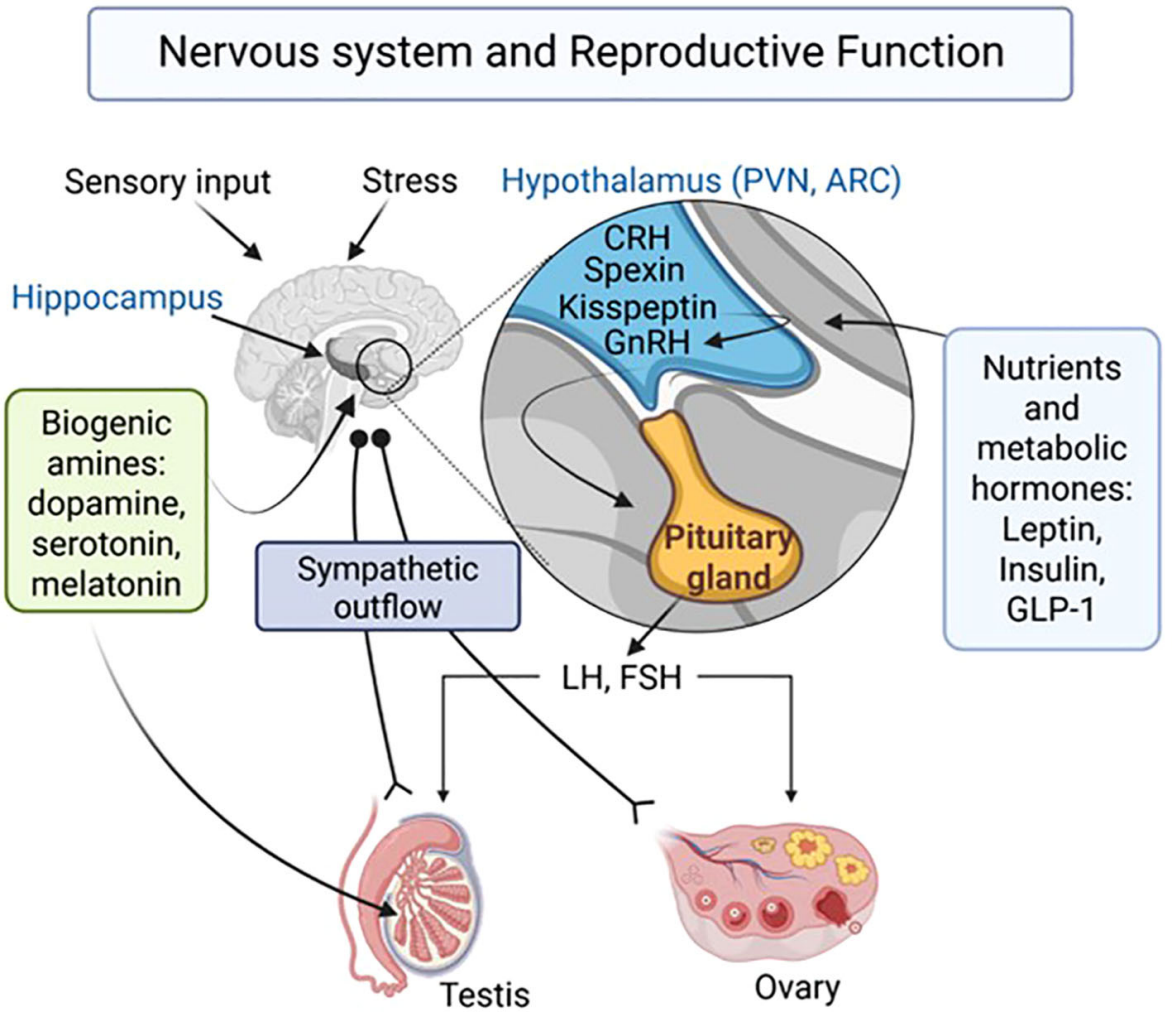
Proposed central and peripheral players and pathways involved in the control of reproductive functions. LH, Luteinizing hormone; FSH, follicle stimulation hormone; CRH, corticotrophin-releasing hormone; GnRH, gonadotrophin-releasing hormone; PVN, Paraventricular nucleus; ARC, arcuate nucleus; GLP-1, Glucagon-like peptide 1. Created in BioRender (https://BioRender.com/rgrf7pt).

Reproductive success is fundamental for the survival of any species. It is regulated at different levels by a multitude of hormonal and neuronal mechanisms. To ensure reproductive success, the integration of sensory information into the hormonal and neural control of the gonads is furthermore mandatory. Some aspects of an integrative partnership between the nervous system and reproduction are addressed in this Research Topic (**Figure 1**), while much more remains to be learned. Such insights will be important and may lead to improved treatment options for reproductive disorders.
